# High *SEC61A1* expression predicts poor outcome of acute myeloid leukemia

**DOI:** 10.1515/med-2024-0944

**Published:** 2024-03-27

**Authors:** Guo Ji, Xiaofei Yang, Jun Li

**Affiliations:** Department of Hematology, Taixing People’s Hospital, Taixing, 225400, Jiangsu, China; National Clinical Research Center for Hematologic Diseases, Jiangsu Institute of Hematology, The First Affiliated Hospital of Soochow University, Suzhou, China; School of Medicine, Southeast University, Institute of Hematology Southeast University, Nanjing, China; Department of Hematology, Taixing People’s Hospital, Changzheng Road No. 1, South Jichuan Road, Taixing, 225400, Jiangsu, China

**Keywords:** *SEC61A1*, AML, biomarker, prognosis

## Abstract

The malfunction of *SEC61A1* has been linked to several types of cancers, but its role in acute myeloid leukemia (AML) remains poorly understood. In this study, we used a series of bioinformatics analysis techniques, including gene expression profiling and proteomic analysis. Our findings were subsequently validated through a series of *in vitro* experiments, such as *SEC61A1* knockdown in cell lines and RT-qPCR. We discovered a significant up-regulation of *SEC61A1* in AML patients compared to healthy controls. AML patients with elevated *SEC61A1* expression exhibited reduced overall survival compared to those with lower expression. Moreover, *SEC61A1* expression emerged as an independent risk factor for predicting the survival of AML patients undergoing allo-HSCT. Our analysis also revealed an association between high *SEC61A1* expression and increased signaling pathways related to cell growth. Our study underscores the importance of *SEC61A1* expression as a novel prognostic indicator for predicting survival among AML patients, while also identifying it as a promising therapeutic target.

## 1 Introduction

Acute myeloid leukemia (AML) stands as a common hematologic malignancy marked by a diverse clonal presence of immature myeloid progenitor cells in both the bone marrow and peripheral blood [1,2], accounting for 80% of newly diagnosed acute leukemia adult patients [1]. Despite noteworthy progress in AML treatment, only 40% of patients below 60 years achieve long-term survival under current therapy [2,3]. Among older patients, survival is significantly worse [3]. Leukemia is classified by two major systems: FAB and WHO. FAB categorizes based on cell types and morphology [4], while WHO considers morphology, immunophenotype, genetics, and clinical features [5]. While FAB was an important first step, WHO offers a more contemporary approach, integrating molecular and immunophenotypic data to enhance therapeutic guidance [5].

Recent advancements in clinical and molecular prognostic markers have markedly enhanced our comprehension of AML biology [5,6]. In addition to traditional markers, the past few years have unveiled novel therapeutic targets and provided insights into identifying individuals who stand to gain the most from specific treatments (such as immune checkpoints [7], macrophage markers [8], chemokine receptors [9], etc.). These breakthroughs are poised to revolutionize the approach to treating individual patients with AML. Therefore, the identification of new molecular markers that can predict survival and serve as treatment targets is of paramount importance.

*SEC61A1* protein belongs to SECY/SEC61-alpha family and is located in the endoplasmic reticulum (ER) membrane [10]. It plays a crucial role in the insertion of membrane polypeptides into the ER and is responsible for the retro-translocation of misfolded proteins to the cytosol for degradation [11]. The aberrant function of the *SEC61A1* protein has been implicated in various human cancers, including head, lung, prostate, and glioblastoma [12–15], and has been targeted as a therapeutic option in multiple myeloma [10]. A recent study has also reported that *SEC61A1* is crucial in mycolactone-dependent apoptosis in AML cells [16]. Nevertheless, the understanding of *SEC61A1* expression in AML remains limited.

In this study, we observed that *SEC61A1* was highly expressed in AML patients compared to healthy controls. High *SEC61A1* expression was associated with poor prognosis in AML and served as a valuable molecular marker for predicting survival.

## 2 Materials and methods

### 2.1 Data collection and processing

In this study, we included 151 AML patients derived from The Cancer Genome Atlas (TCGA) dataset [17], with ages ranging from 18 to 88 years. These patients were diagnosed and treated at Washington University between 2001 and 2010 [17]. Following the NCCN guideline, all AML patients underwent the standard induction and consolidation regimen. As stated by the teams, written consents for the study were all available, following the Declaration of Helsinki [17]. Samples of peripheral blood were derived at the time point of diagnosis. Clinical information on survival, baseline characteristics, gene mutation profile, and expression profile could all be downloaded from the TCGA. Additionally, AML-M3 patients were excluded from the study.

Other AML datasets were collected from Gene Expression Omnibus (GEO) (GEO accession numbers: GSE7186 [18], GSE13159 [19], GSE22778 [20], GSE12417 [21]). Written consent for the treatment and the study was also available, as stated by the authors. Microarray data, as well as clinical characteristics, could all be downloaded from the GEO dataset. Proteomic expression data were derived from the Proteomic Data Commons (PDC, National Cancer Institute) (PDC Study Identifier: PDC000477, Project ID: Proteogenomic Translational Research Centers [PTRC]) [22].

### 2.2 Univariate and multivariate Cox proportional hazards regression model of overall survival with patients’ features (age, sex, mutations, etc.)

The association between survival time with patients’ clinical features was investigated by conducting a univariate and multivariate Cox proportional hazards regression model. Clinical features, including age, sex, white blood cell, platelet counting of peripheral blood, and gene mutation were included in univariate Cox analysis. The hazard ratio (HR) is defined as the ratio of the probability of the event occurring in the exposed group versus the control (non-exposed) group. The inclusion criteria of multi-Cox analysis must meet at least one of the following items: (1) a *P*-value of univariate Cox analysis result ≤0.20 and (2) clinically recognized that it can have a significant impact on the prognosis. *P*-value ≤0.05 in multivariate Cox analysis, indicating an independent impact on patient’s survival time. The concordance index (c-index) is a metric to evaluate the predictions made by an algorithm. It is defined as the proportion of concordant pairs divided by the total number of possible evaluation pairs (0.5 < c-index ≤ 0.7, indicating low predictive accuracy; 0.71 ≤ c-index ≤ 0.9, indicating middle predictive accuracy; c-index > 0.9, indicating high predictive accuracy; *P*-value <0.05, indicating that the hazard model is significant).

### 2.3 Reverse transcription quantitative PCR (RT‑qPCR)

To extract the total RNA from AML cell lines, we used the Trizol agent (Vazyme Biotech Co., Ltd) following the manufacturer’s protocol. Then HiScript 1st Strand cDNA Synthesis Kit (Vazyme Biotech Co., Ltd) was used to reverse transcribe the extracted mRNA into cDNA. The real-time PCR was performed on a CFX96 TOUCH analysis system (Bio-rad, USA), using ChamQ Universal SYBR qPCR 1Master Mix (Vazyme Biotech Co., Ltd). In this study, we used the following amplification conditions of qPCR: 95°C of pre-denaturation for 30 s, 40 cycles of 95°C of denaturation for 5 s, and 60°C of extension for 30 s. The primer sequences were as follows:

*SEC61A1* Forward: 5′- GAAGGAGCAGCAGATGGTGATGAG-3′, Reverse: 5′- GGAAGTCAGCCAGGACCGAGAGAG-3′.

*FLT3* Forward: 5′- ACCTCAAGTGCTCGCAGAAGCA-3′, Reverse: 5′-GTTAGCCTTTCTATTCCAGACTCC.

*GAPDH* Forward: 5′-GCAAATTCCATGGCACCGT-3′, Reverse: 5′- GACTCCACGACGTACTCAGC-3′.

The relative expression levels of the target genes were calculated by the 2^−ΔΔCt^ method.

### 2.4 Cell lines

The U937 and MV4-11 cell lines were grown in a 5% CO_2_ atmosphere at 37°C. U937 cells were grown in RPMI-1640 (Gibco, Beijing, China) supplemented with 10% fetal bovine serum (FBS) (Gibco, Beijing, China), MV4-11 cells were grown in IMDM (Gibco, Beijing, China) supplemented with 10% FBS.

### 2.5 Plasmid construction, lentiviral transduction, and target gene knockdown

Following the manufacturer’s instruction, we constructed lentiviral shRNA plasmids for *SEC61A1* by subcloning the shRNA oligos into the lentiviral shRNA vector with an IRES GFP (pLV3ltr-ZsGreen-Puro-U6) (Corues Biotechnology, China). The shRNA oligos design for *SEC61A1* is listed in Table S1.

Following the manufacturer’s instructions, 293T cells were transfected with the indicated viral plasmid and psPAX2 and pMD2.G (packaging plasmids) (Corues Biotechnology, China), via Exfect Transfection Reagent (Vazyme Biotech Co., Ltd, China, No. T101-01). After 48 and 72 h post-transfection, viral supernatant was collected and filtered with a 45 μM filter. AML cells were therefore transduced with the resulting lentivirus. HitransG P (KeyGen BioTECH, China) was added to increase the transduction efficiency. After 48 h transduction, 1 µg/mL of puromycin (InvivoGen, CAS.58-58-2) was added into the infected cells for stable expressing cell selection. Via flow cytometry, the efficiency of transduction was monitored by GFP (+) cells, more than 95% of GFP (+) cells were used for knockdown efficiency and further *in vitro* experiments.

### 2.6 Bioinformatic analysis of RNA sequencing data

In this study, all bioinformatic analysis was conducted on the R platform and Perl script, R package SVA was used to remove the batch effects and recover the biological signal in the data when integrating different GPL platforms from a common GEO series (GSE22778 contains nine platforms, including GPL8650, GPL8651, GPL8652, GPL8653, GPL8654, GPL10105, GPL10106, GPL10107, GPL10108, *n* = 185; GSE12417 contains three platforms, including GPL96, GPL97, GPL570, *n* = 405). R package edgeR was used to conduct the differential expression analysis of the two cohorts (*SEC61A1* ^high^ and *SEC61A1* ^low^ cohort). All R scripts used for data analysis in this study are publicly available from GitHub (https://github.com). We used Benjamini and Hochberg’s method to control the false discovery rate. Genes with a fold change over 1.5 (adjusted *P*-value <0.01) found by edgeR were considered as differentially expressed. Analysis of specific signaling pathways was conducted on Gene Set Enrichment Analysis (GSEA) platform.

This study utilized publicly accessible data from TCGA and GEO databases. These databases contain genomic and expression data from patients worldwide and are anonymized, devoid of directly identifiable personal information.

### 2.7 Statistical analysis

Data were visualized and analyzed using STATA 16.0 (StataCorp 2019) and Prism 8.0 (GraphPad Prism software). In this study, data were presented as the mean ± SD (normalized distribution) or median (IQR) (skewed distribution). *t-*test was used to compare two groups, using Prism 8.0 software. Kaplan–Meier method was used to conduct the survival analysis.


**Ethical statement:** The collection and sharing of data in TCGA and GEO databases adhere to stringent ethical guidelines and regulations, encompassing patient informed consent, privacy protection, and data security. The original data collection received approval from the respective ethics committees, and during the sharing process, any potentially identifying information was removed. The use of data in this study aligns with the data usage policies of TCGA and GEO, posing no potential risks to the privacy and rights of participants. It is explicitly stated that this study does not involve direct experimentation on human or animal subjects; rather, it analyzes publicly available, lawfully obtained genomic and expression data.

## 3 Results

### 3.1 Overexpression of *SEC61A1* accompanied by the poor prognosis of AML

**Figure 1:**
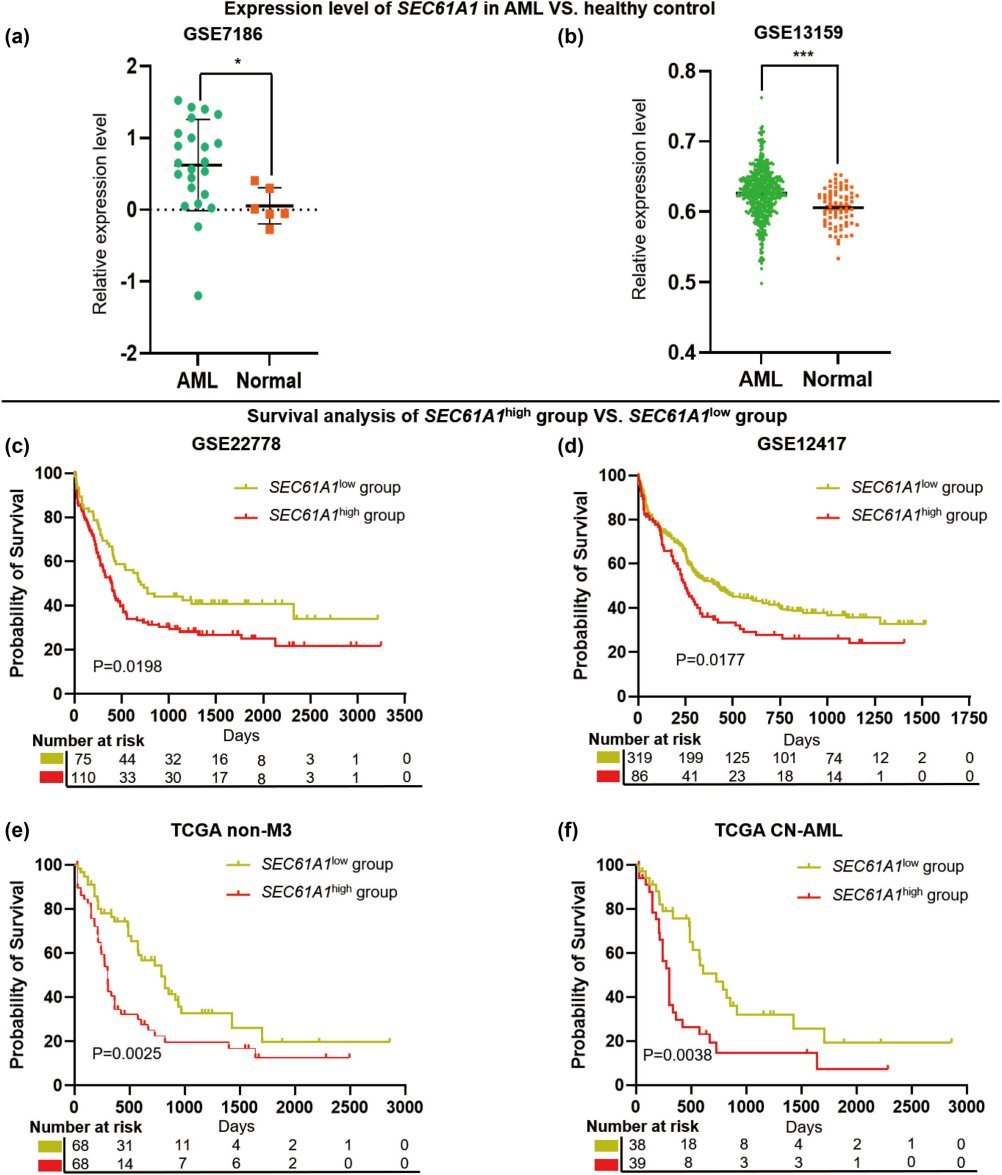
High *SEC61A1* expression predicts adverse clinical outcome among AML patients. (a) and (b) Expression level of *SEC61A1* in AML are higher, compared with healthy control (samples of GSE7186 and GSE13159 were both derived from bone marrow; *P < 0.05; ***P < 0.0001); (c)–(f) OS analysis of *SEC61A1* expression in AML patients, high expression of *SEC61A1* predicts poor prognosis in GSE122778 (*SEC61A1* ^high^ group = 110, *SEC61A1* ^low^ group = 75), GSE12417 (*SEC61A1* ^high^ group = 86, *SEC61A1* ^low^ group = 319), TCGA-non M3 dataset (*SEC61A1* ^high^ group = 68, *SEC61A1* ^low^ group = 68), and TCGA-CN AML dataset (*SEC61A1* ^high^ group = 39, *SEC61A1* ^low^ group = 38).

Two datasets derived from GEO datasets (GSE7186 and GSE13159) were used to compare the expression level of *SEC61A1* between AML and healthy cohorts. The results denoted that the expression of *SEC61A1* was significantly increased in AML patients, compared to controls (GSE7186, *P* = 0.0435; GSE13159, *P* < 0.0001) (Figure 1a and b).

We further hypothesized that high *SEC61A1* expression is associated with poor prognosis in AML patients. Hence, we used three datasets to confirm this finding. The results denoted that high *SEC61A1* expression predicts unfavorable prognosis in overall survival in AML (*SEC61A1* ^high^ vs *SEC61A1* ^low^ AML cohort, GSE22778, *P* = 0.0198; GSE12417, *P* = 0.0177; TCGA [excluding AML-M3], *P* = 0.0025; TCGA CN-AML, *P* = 0.0038) (Figure 1c–f).

**Figure 2:**
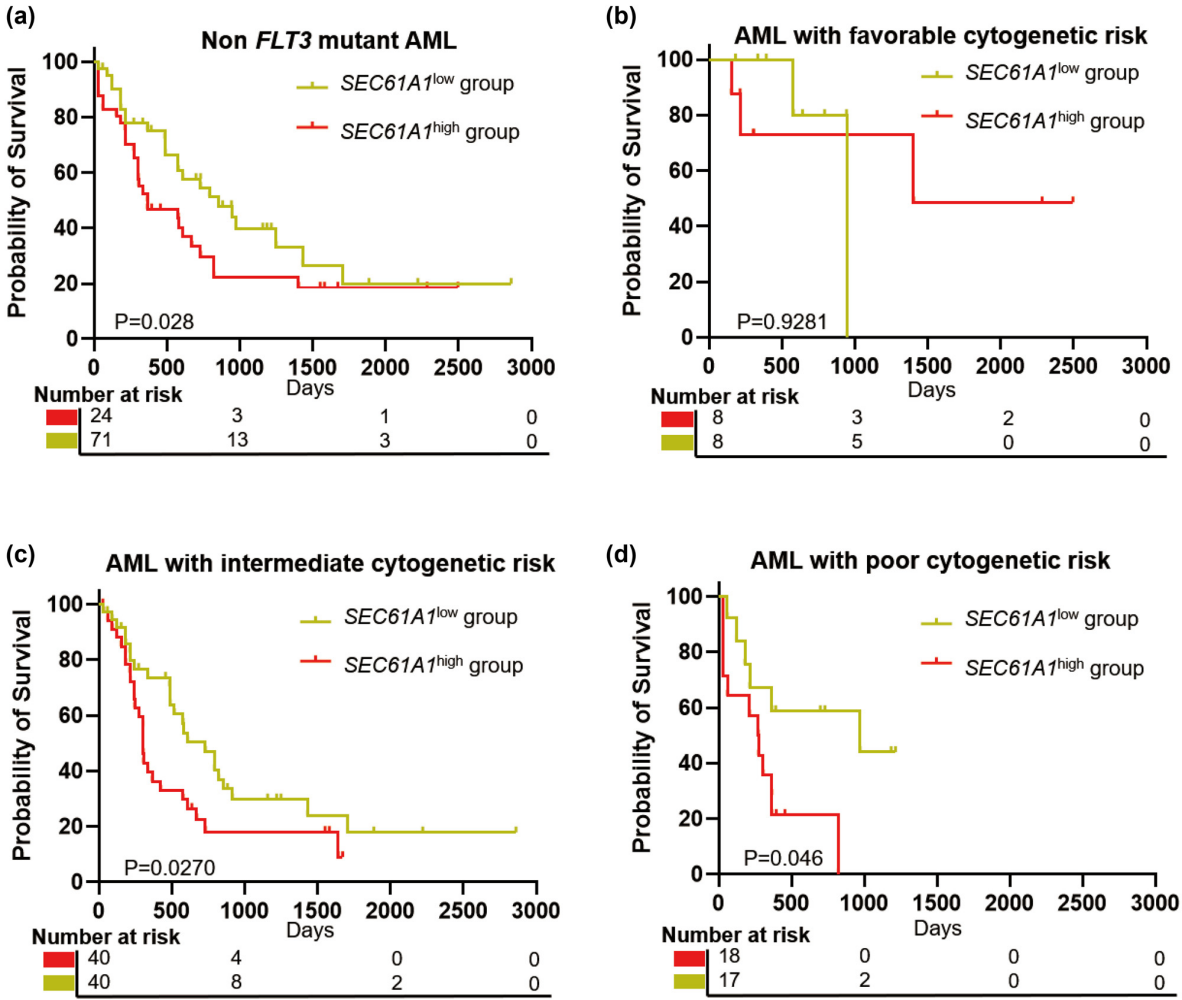
High *SEC61A1* expression predicts adverse clinical outcome among AML patients. (a) OS analysis of *SEC61A1* expression in non-FLT3 mutant AML patients, high expression of *SEC61A1* predicts poor prognosis. (b)–(d) OS analysis of *SEC61A1* expression in favorable cytogenetic risk (b), intermediate risk (c), and poor risk (d).

In addition, the expression of *SEC61A1* serves as an indicator for poor OS in non-*FLT3* mutant AML patients (TCGA-AML, *P* = 0.028) (Figure 2a), with *SEC61A1* demonstrating notably dismal survival outcomes in the intermediate to high-risk groups (Figure 2b–d). Intriguingly, upon downregulating the expression of *SEC61A1* in AML cell lines (Figures S1a, b and S2), we observed a simultaneous reduction in *FLT3* expression (Figure S1a and b). Furthermore, we identified a positive correlation between the expression of *SEC61A1* and *FLT3* in AML (*P* = 0.0068, *R* = 0.21) (Figure S3). Further categorizing AML patients based on the expression levels of *SEC61A1* and *FLT3*, we classified them into three groups (*SEC61A1* ^low^ with *FLT3* ^low^, *SEC61A1* ^low^ and *FLT3* ^high^ or *SEC61A1* ^high^ with *FLT3* ^low^, and *SEC61A1* ^high^ with *FLT3* ^high^). Notably, the *SEC61A1* ^low^ with *FLT3* ^low^ group exhibited the most favorable survival trend among the three, while the *SEC61A1* ^high^ with *FLT3* ^high^ group demonstrated the poorest survival outcome (Figure S3).

### 3.2 Baseline characters of *SEC61A1* ^high^ and *SEC61A1* ^low^ AML cohort of TCGA dataset

We conducted a comparison of baseline clinical characteristics and gene mutations between *SEC61A1* ^high^ and *SEC61A1* ^low^ AML cohorts using the TCGA dataset. However, no significant differences were observed in age, gender, race, WBC count, platelet count, BM blast percentage, and gene mutations (Table 1). Nevertheless, we discovered that the *SEC61A1* ^high^ cohort exhibited a higher proportion of *FLT3* mutation (*P* = 0.005) (Table 1).

**Table 1:** Baseline clinical characteristics of non-M3-AML patients from the TCGA

Characteristic		*SEC61A1* ^low^	*SEC61A1* ^high^	*P*
Total		66	67	
Cytogenetic risk, *n* (%)	Favorable	8 (6.1%)	8 (6.1%)	0.965
	Intermediate	41 (31.3%)	39 (29.8%)	
	Poor	17 (13%)	18 (13.7%)	
Chromosome abnormality, *n* (%)	8	7 (5.9%)	7 (5.9%)	0.992
	Complex over three distinct abnormalities	1 (0.8%)	2 (1.7%)	
	del(5q)/5q-	2 (1.7%)	3 (2.5%)	
	del(7q)/7q-	7 (5.9%)	6 (5.1%)	
	inv(16)	4 (3.4%)	3 (2.5%)	
	Normal	36 (30.5%)	31 (26.3%)	
	*t*(8;21)	3 (2.5%)	4 (3.4%)	
	*t*(9;11)	1 (0.8%)	1 (0.8%)	
Gender, *n* (%)	Female	36 (27.1%)	25 (18.8%)	0.056
	Male	30 (22.6%)	42 (31.6%)	
FAB classification, *n* (%)	M0	8 (6.0%)	6 (4.5%)	0.664
	M1	14 (10.5%)	21 (15.8%)	
	M2	18 (13.5%)	20 (15%)	
	M4	12 (9%)	15 (11.3%)	
	M5	7 (5.3%)	8 (6%)	
	M6	0 (0%)	2 (1.5%)	
	M7	0 (0%)	1 (0.8%)	
	Not classified	1 (0.8%)	0 (0%)	
Race, *n* (%)	Asian	0 (0%)	1 (0.8%)	0.763
	Black or African American	5 (3.8%)	6 (4.5%)	
	White	61 (46.2%)	59 (44.7%)	
Age, median (IQR)		54 (41.25, 67)	60 (44.5, 67)	0.216
Platelet counting (×10^9^/L), median (IQR)		52 (28.5, 87)	43 (31, 88)	0.768
WBC counting (×10^9^/L), median (IQR)		20.5 (6, 52.75)	22 (5.5, 46)	0.932
BM blast (%), median (IQR)		46.5 (10, 71)	41 (11.5, 60)	0.659
Hemoglobin (g/L), *n* (%)		9(9,10)	9(9,11)	0.7695
Treatment	“3 + 7” regimen	52	50	0.570
HSCT, *n* (%)	allo-HSCT	35 (26.3%)	26 (19.5%)	0.141
	No allo-HSCT	31 (23.3%)	41 (30.8%)	
*PTPN11* mutant		4 (3%)	2 (1.5%)	0.441
*CEBPA* mutant		5 (3.8%)	8 (6%)	0.579
*TET2* mutant		5 (3.8%)	7 (5.3%)	0.783
*FLT3* mutant		11 (8.3%)	27 (20.3%)	0.005
*TP53* mutant		5 (3.8%)	6 (4.5%)	0.999
*EZH2* mutant		0 (0%)	2 (1.5%)	0.496
*NRAS* mutant		4 (3%)	3 (2.3%)	0.718
*NPM1* mutant		16 (12%)	22 (16.5%)	0.366
*DNMT3A* mutant		16 (12%)	20 (15%)	0.594
*RUNX1* mutant		8 (6%)	6 (4.5%)	0.755
*GATA2* mutant		1 (0.8%)	1 (0.8%)	0.999

*Abbreviations: FAB classification: French–American–British classification; allo-HSCT: allogeneic hematopoietic stem cell transplantation; “3 + 7” regimen includes: daunorubicin plus cytarabine.

### 3.3 Cox proportional hazard model analysis of *SEC61A1* expression in CN-AML patients

To gain a better understanding of the correlations between *SEC61A1* expression and overall survival, we conducted univariate Cox hazard analysis for TCGA AML patients (excluding M3-AML) based on whether they received allo-HSCT. The included variables were *SEC61A1* expression, age, WBC count, BM-blast, hemoglobin, platelet count, cytogenetic risk, and gene mutations (detailed list in Tables S2 and S3, mutation vs. wild type). In patients who did not receive allo-HSCT, the results unveiled that age (HR = 1.029, *P* = 0.004), intermediate cytogenetic risk (HR = 4.247, *P* = 0.016), poor cytogenetic risk (HR = 20.555, *P* < 0.001), *TP53* mutation (HR = 3.907, *P* < 0.001), and *DNMT3A* mutation (HR = 1.939, *P* = 0.033) were identified as risk factors. However, other variables showed no statistical significance (Table S2). Meanwhile, for patients who received allo-HSCT, the results indicated that *SEC61A1* expression was a significant risk factor (HR = 16.635, *P* = 0.006) (Table S3).

**Figure 3:**
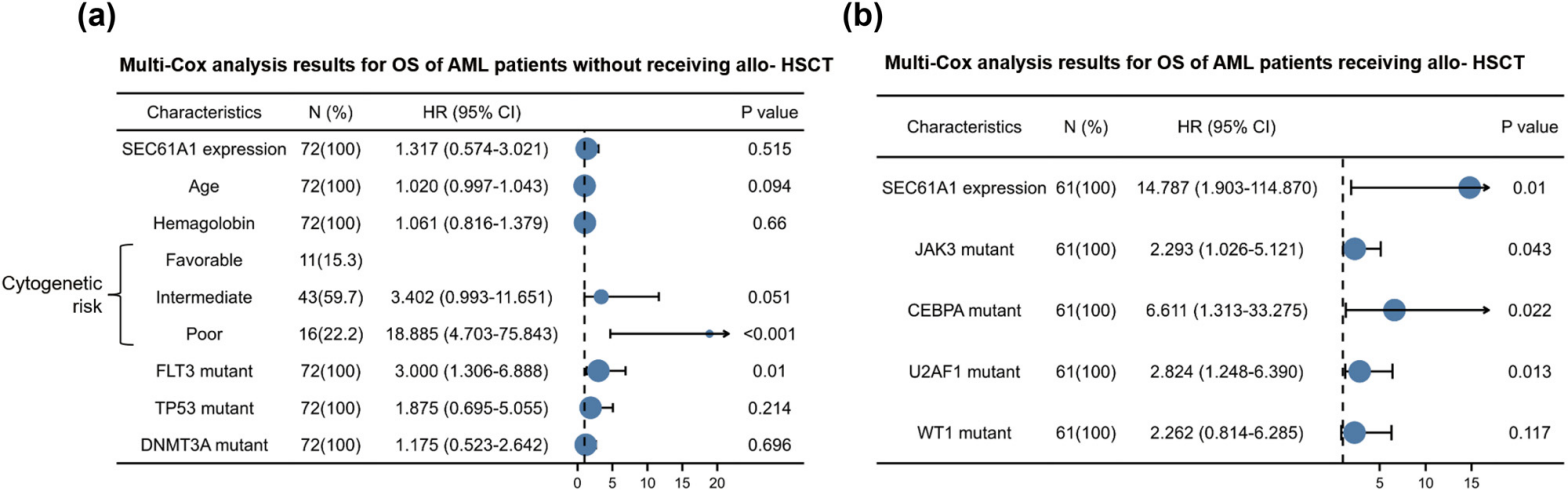
Forest plot of multivariate COX hazard regression model (TCGA AML-non M3). (a) Forest plot of multivariate COX hazard regression analysis among AML patients without receiving allo-HSCT. (b) Forest plot of multivariate COX hazard regression analysis among AML patients receiving allo-HSCT.

We further conducted a multivariate Cox hazard analysis for overall survival. Parameters with *P* values less than 0.2 during univariate Cox hazard analysis were included in the hazard model. The results revealed that poor cytogenetic risk (HR = 18.885, *P* < 0.001) and *FLT3* mutation (HR = 3.0, *P* = 0.01) were independent risk factors for patients who did not undergo allo-HSCT (Figure 3a) (*P* < 0.05). Among patients who received allo-HSCT, *SEC61A1* expression (HR = 14.787, *P* = 0.01), *JAK3* mutation (HR = 2.293, *P* = 0.043), *CEBPA* mutation (HR = 6.611, *P* = 0.022), and *U2AF1* mutation (HR = 2.824, *P* = 0.013) were identified as independent risk factors. Furthermore, high *SEC61A1* expression was found to be the highest risk factor among patients who received allo-HSCT (Figure 3b). These findings indicate that high *SEC61A1* expression is an independent risk factor for AML patients udnergoing allo-HSCT.

### 3.4 Genome-wide expression profile associated with *SEC61A1* expression

**Figure 4:**
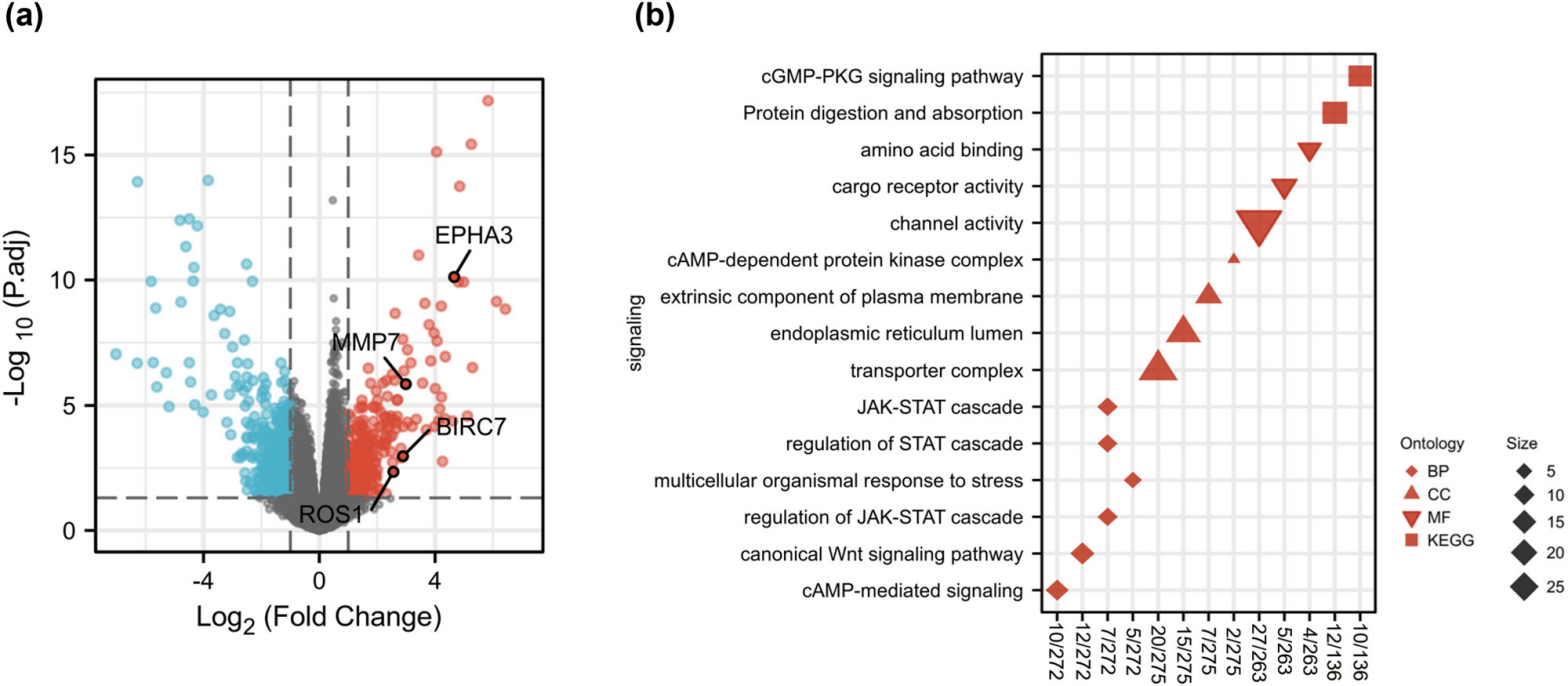
Genome wide expression profile associated with *SEC61A1* expression. (a) Volcano plot of DEGs (*SEC61A1* ^high^ vs *SEC61A1* ^low^ AML cohort, |log_2_FC| > 2, *P*-value <0.05). (b) GO and KEGG analysis result of the DEGs.

**Figure 5:**
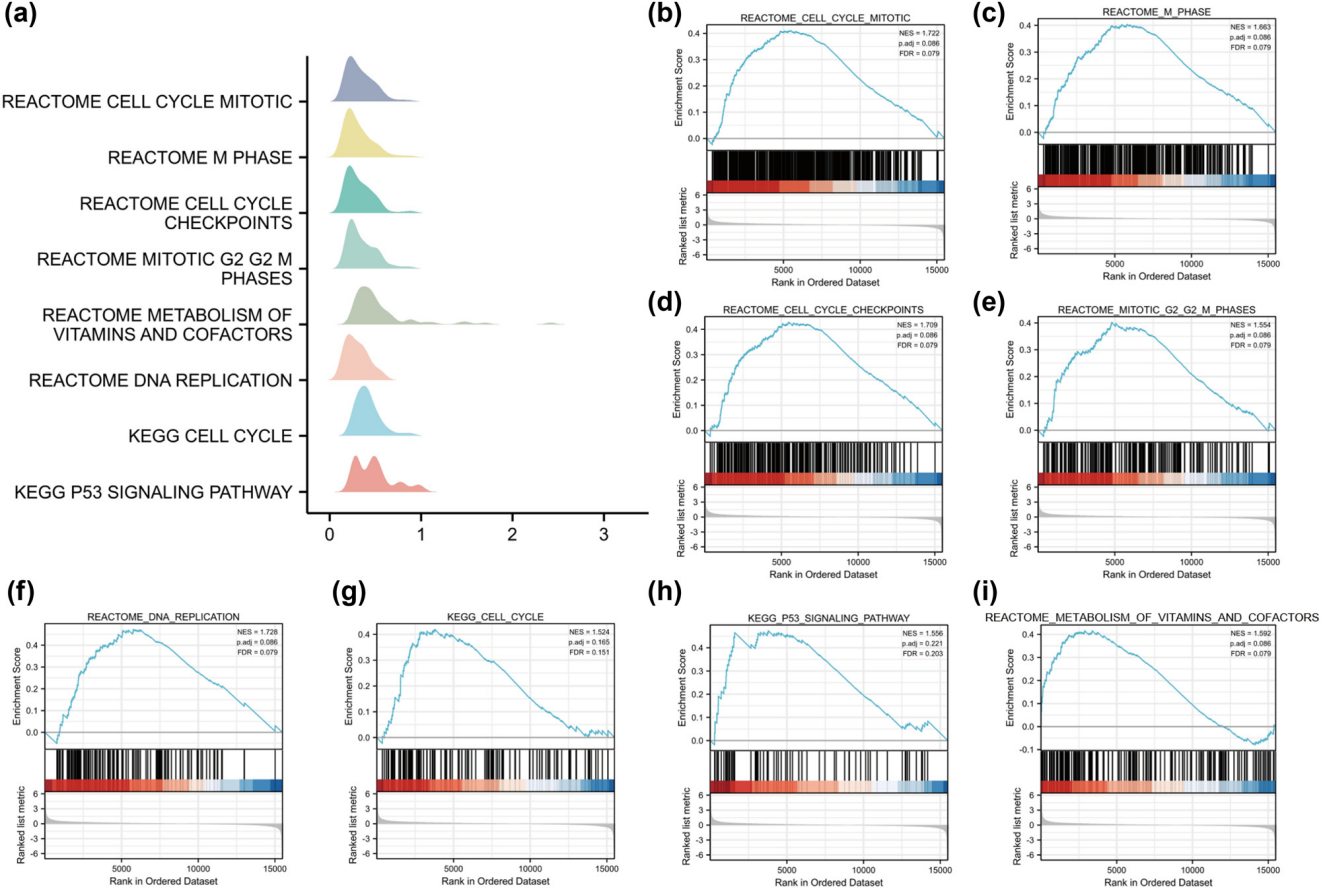
GSEA analysis result of signaling pathways associated with *SEC61A1* expression. (a)–(i) Top eight enriched signaling pathways by GSEA analysis are listed.

To illustrate the underlying molecular mechanism associated with high *SEC61A1* expression, we further explored the gene expression profile from the TCGA database. By utilizing R software, we identified 162 differentially expressed genes (DEGs) (*SEC61A1* ^high^ vs *SEC61A1* ^low^ AML cohort, |log_2_FC| > 2, *P*-value <0.05), including 83 upregulated and 79 downregulated genes (Figure 4a). Notably, *EPHA3, MMP7, BIRC7*, and *ROS1* were found to be upregulated (Figure 4a). Gene ontology (GO) and Kyoto Encyclopedia of Genes and Genomes (KEGG) analysis revealed significant enrichment in the cGMP-PKG signaling pathway and JAK-STAT signaling pathway (Figure 4b). We also performed GSEA analysis to investigate the signaling pathways associated with high *SEC61A1* expression. Interestingly, several cell cycle-associated signaling pathways, such as Reactome cell cycle mitotic, Reactome Mphase, and Reactome cell cycle checkpoints, were found to be enriched in the high *SEC61A1* expression cohort (Figure 5a–i).

**Figure 6:**
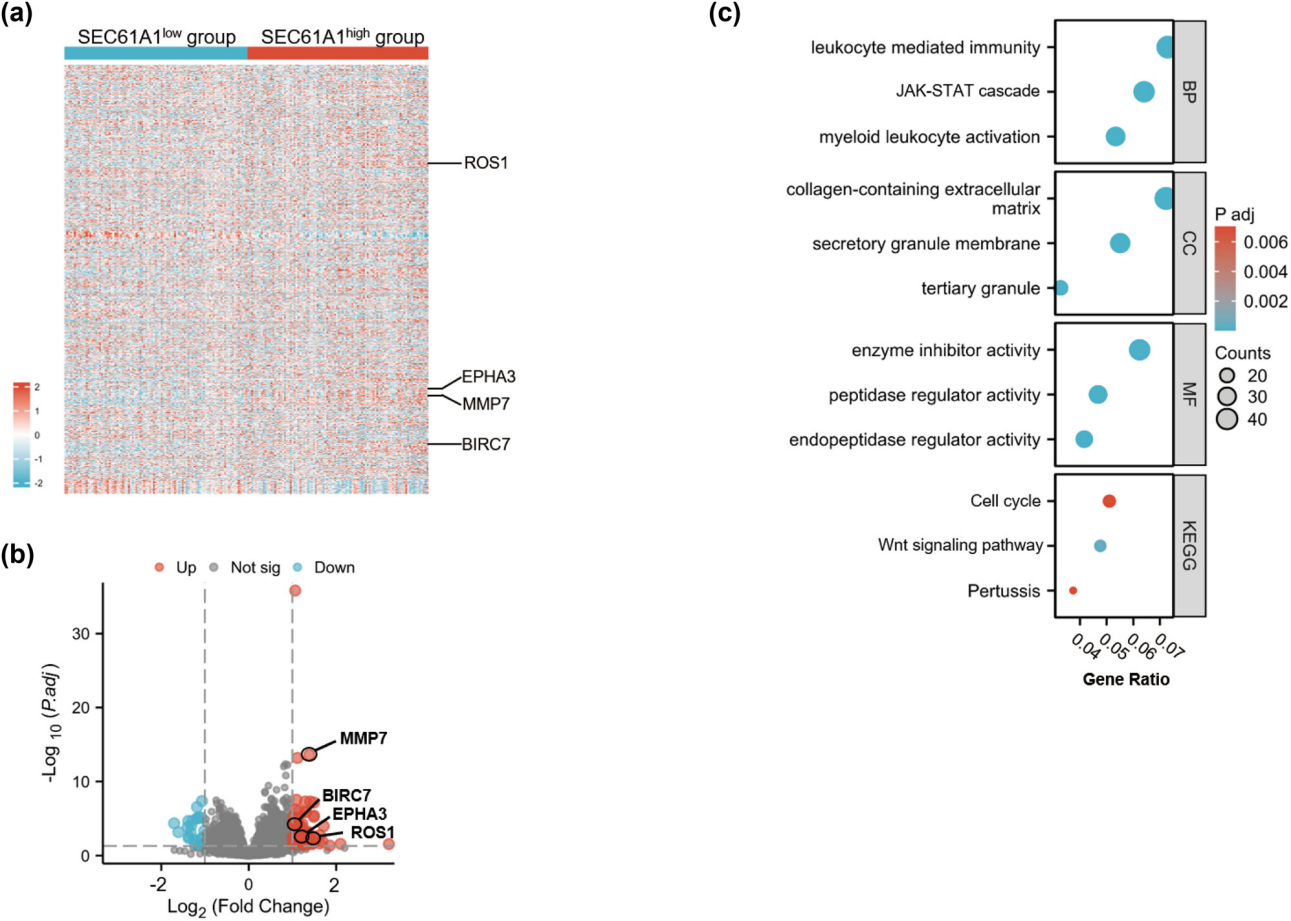
Proteomic analysis result associated with *SEC61A1* expression. (a) Heatmap of differentially expressed proteins associated with *SEC61A1* expression. (b) Volcano plot of differentially expressed proteins (*SEC61A1* ^high^ vs *SEC61A1* ^low^ AML cohort, |log_2_FC| > 2, *P*-value <0.05). (c) Top enriched signaling pathways by GO and KEGG analysis are listed.

To validate our aforementioned findings, we conducted further investigation into proteomic expression patterns associated with *SEC61A1* expression in AML patients using data sourced from the PDC database. A cohort comprising 189 AML patients was included in our analysis. As illustrated in Figure 6a and b, EPHA3, MMP7, BIRC7, and ROS1 exhibited significant upregulation in the *SEC61A1* ^high^ (*n* = 95) compared to the *SEC61A1* ^low^ (*n* = 94) AML cohort, with |log_2_FC| > 2 and a *P*-value <0.05. Additionally, our GO and KEGG analysis revealed pronounced enrichment in the JAK-STAT signaling pathway. Notably, we observed significant enrichment in “leukocyte mediated immunity” and “myeloid leukocyte activation” associated with *SEC61A1* expression.

## 4 Discussion

*SEC61A1* plays a crucial role in the biogenesis of most secreted and transmembrane proteins [11,23]. This process is fundamental to the proper folding, modification, and transport of proteins destined for secretion or integration into cellular membranes [11,23]. Previous studies have indicated that targeting *SEC61A1* enhances sensitivity to anticancer drugs in various hematological malignancies [24–26]. The primary mechanism underlying this effect involves the augmentation of ER stress [26]. Moreover, *SEC61A1* has been shown to sensitize AML cells to other drugs by inducing apoptosis [10]. Therefore, investigating the significance of *SEC61A1* expression in AML is of great importance.

Traditionally, disease classification relied heavily on pathological and cytological features. However, with a deeper understanding of cancer genetics and proteins, researchers have identified unique molecular markers that aid in the precise categorization of disease subtypes [6]. Furthermore, the emergence of new drugs based on these molecular markers signifies a shift toward individualized therapy. The principles of personalized treatment involve tailoring therapeutic approaches based on a patient’s specific molecular and genetic characteristics [6]. This customization enhances treatment efficacy while minimizing adverse effects on patients.

In this study, we have identified *SEC61A1* expression as a novel prognostic marker for AML. Our analysis unveiled a significantly upregulation of *SEC61A1* in AML patients compared to healthy controls. Furthermore, higher *SEC61A1* expression was found to be associated with shorter overall survival for non-M3 AML patients, especially among those with intermediate/poor risk. Importantly, we demonstrated that *SEC61A1* expression was an independent risk factor for OS among AML patients receiving allo-HSCT, suggesting that targeting *SEC61A1* may be a valuable strategy for preventing disease relapse after allo-HSCT. Another interesting finding is that we also observed a higher incidence of *FLT3* mutations in the *SEC61A1* high-expression cohort. *FLT3* mutations are relatively common in AML patients, accounting for approximately 30%, and are associated with a poorer prognosis [27]. Most FLT3 proteins undergo unfolding within the ER lumen [28], leading to increased ER stress [29]. Given that *SEC61A1* serves as a crucial regulator of ER stress [23], its potential role in maintaining ER homeostasis in AML cells with *FLT3* mutations could be significant. Further research is warranted to delve into the intricacies of this relationship and explore additional details in future studies. Above findings have significant implications for the management and treatment of AML patients.

Furthermore, we identified *SEC61A1* as a novel prognostic marker for AML and investigated its potential molecular mechanisms. We found that high expression of *SEC61A1* was associated with upregulation of *EPHA3, MMP7, BIRC7*, and *ROS1*, all of which have been reported as prognostic markers or therapeutic targets in hematological malignancies [30–33]. Furthermore, we explored the correlation between high *SEC61A1* expression and several oncogenic signaling pathways. The results revealed that the high *SEC61A1* expression was linked to enrichment of the cGMP-PKG and JAK-STAT signaling pathways. It is noteworthy that an overactive cGMP-PKG signaling pathway was reported with anti-apoptotic activity [34], whereas JAK-STAT signaling pathway was reported to be implicated in cell growth and cycle arrest in AML [35]. In addition, our GSEA analysis also supported the association between *SEC61A1* expression and cell cycle activity in AML, including “cell cycle M phase, cell cycle G2/M phase, cell cycle checkpoint,” which are indicative of the cell growth dynamics of leukemic cells. Consequently, our proteomic analysis revealed enrichment of the JAK-STAT signaling pathway and cell cycle pathways associated with *SEC61A1* expression. These findings serve to affirm the correlation between elevated *SEC61A1* expression and activation of oncogenic signaling pathways.

## 5 Conclusions

In summary, our findings propose *SEC61A1* as a promising prognostic marker for assessing the survival outcomes of AML patients. The preliminary insights gleaned from our study regarding this novel marker hold potential for clinical application in the future. We anticipate that our results will catalyze further investigation in this domain, facilitating advancements in AML prognostication and treatment strategies.

## Supplementary Material

supplementary material
