# Supplementary material for: High SEC61A1 expression predicts poor outcome of acute myeloid leukemia
Source: Open Med (Wars). 2024 Mar 27;19(1):20240944. doi: 10.1515/med-2024-0944 (PMC10997032; doi:10.1515/med-2024-0944)
Supplement: supplementary material [file med-2024-0944-sm.pdf]

Supplementary material

Table S1: Oligo design for shRNA of *SEC61A1*

	Oligo design
SEC61A1-iF	gatccGCAACTAACATCTGTGAAACCtcaagagGGTTTCACAGATGTTAGTTGCtttttt
SEC61A1-iR	aattaaaaaaGCAACTAACATCTGTGAAACCctcttgaGGTTTCACAGATGTTAGTTGCg

Table S2: Uni-Cox analysis results for OS of AML patients receiving allo- HSCT

**Characteristics**		**Total(*N*)**	**Univariate analysis**	
			**Hazard ratio (95% CI)**	***P* value**
SEC61A1		72	1.957 (0.837–4.574)	0.121
Cytogenetic risk	Favorable	11	Reference	
	Intermediate	43	4.247 (1.303–13.840)	**0.016**
	Poor	16	20.555 (5.733–73.692)	<**0.001**
Age		72	1.029 (1.009–1.049)	**0.004**
Platelet counting		72	0.999 (0.992–1.005)	0.640
WBC counting		72	1.002 (0.996–1.008)	0.564
Hemaglobin		72	1.151 (0.936–1.415)	0.182
BM blast percentage		72	0.998 (0.990–1.007)	0.727
CEBPA	Wild type	67	Reference	
	Mutant	5	1.122 (0.405–3.111)	0.825
TET2	Wild type	63	Reference	
	Mutant	9	0.846 (0.383–1.873)	0.681
FLT3	Wild type	51	Reference	
	Mutant	21	1.694 (0.941–3.049)	0.079
TP53	Wild type	63	Reference	
	Mutant	9	3.907 (1.852–8.240)	<**0.001**
U2AF1	Wild type	68	Reference	
	Mutant	4	0.752 (0.234–2.417)	0.632
SMC1A	Wild type	68	Reference	
	Mutant	4	1.331 (0.479–3.704)	0.583
NRAS	Wild type	68	Reference	
	Mutant	4	0.752 (0.182–3.101)	0.694
IDH2	Wild type	64	Reference	
	Mutant	8	0.781 (0.366–1.664)	0.521
IDH1	Wild type	66	Reference	
	Mutant	6	1.195 (0.429–3.327)	0.733
NPM1	Wild type	50	Reference	
	Mutant	22	1.071 (0.606–1.895)	0.813
DNMT3A	Wild type	51	Reference	
	Mutant	21	1.939 (1.056–3.560)	**0.033**
RUNX1	Wild type	65	Reference	
	Mutant	7	1.410 (0.635–3.131)	0.398
SMC3	Wild type	68	Reference	
	Mutant	4	1.304 (0.404–4.212)	0.657

Table S3: Uni-Cox analysis results for OS of AML patients without receiving allo- HSCT

**Characteristics**		**Total(*N*)**	**Univariate analysis**	
			**Hazard ratio (95% CI)**	***P* value**
SEC61A1		61	16.635 (2.285–121.114)	**0.006**
Cytogenetic risk	Favorable	5	Reference	
	Intermediate	37	1.831 (0.427–7.853)	0.416
	Poor	19	2.040 (0.444–9.371)	0.360
Age		61	1.003 (0.979–1.028)	0.789
Platelet counting		61	1.001 (0.997–1.006)	0.585
Leukocyte counting		61	1.004 (0.997–1.011)	0.278
Hemaglobin		60	1.048 (0.830–1.322)	0.696
BM blast percentage		61	1.003 (0.992–1.015)	0.585
PTPN11	Wild type	57	Reference	
	Mutant	4	1.212 (0.367–4.000)	0.752
SETBP1	Wild type	53	Reference	
	Mutant	8	0.964 (0.372–2.499)	0.941
JAK2	Wild type	58	Reference	
	Mutant	3	0.845 (0.200–3.564)	0.818
JAK3	Wild type	44	Reference	
	Mutant	17	1.922 (0.928–3.980)	0.079
CEBPA	Wild type	59	Reference	
	Mutant	2	3.762 (0.852–16.613)	0.080
RAD21	Wild type	53	Reference	
	Mutant	8	1.340 (0.551–3.258)	0.519
TET2	Wild type	58	Reference	
	Mutant	3	0.315 (0.043–2.326)	0.258
FLT3	Wild type	53	Reference	
	Mutant	8	0.953 (0.287–3.161)	0.937
TP53	Wild type	53	Reference	
	Mutant	8	0.537 (0.186–1.546)	0.249
EZH2	Wild type	45	Reference	
	Mutant	16	0.845 (0.391–1.826)	0.668
U2AF1	Wild type	46	Reference	
	Mutant	15	1.835 (0.855–3.939)	0.119
WT1	Wild type	54	Reference	
	Mutant	7	2.112 (0.793–5.624)	0.135

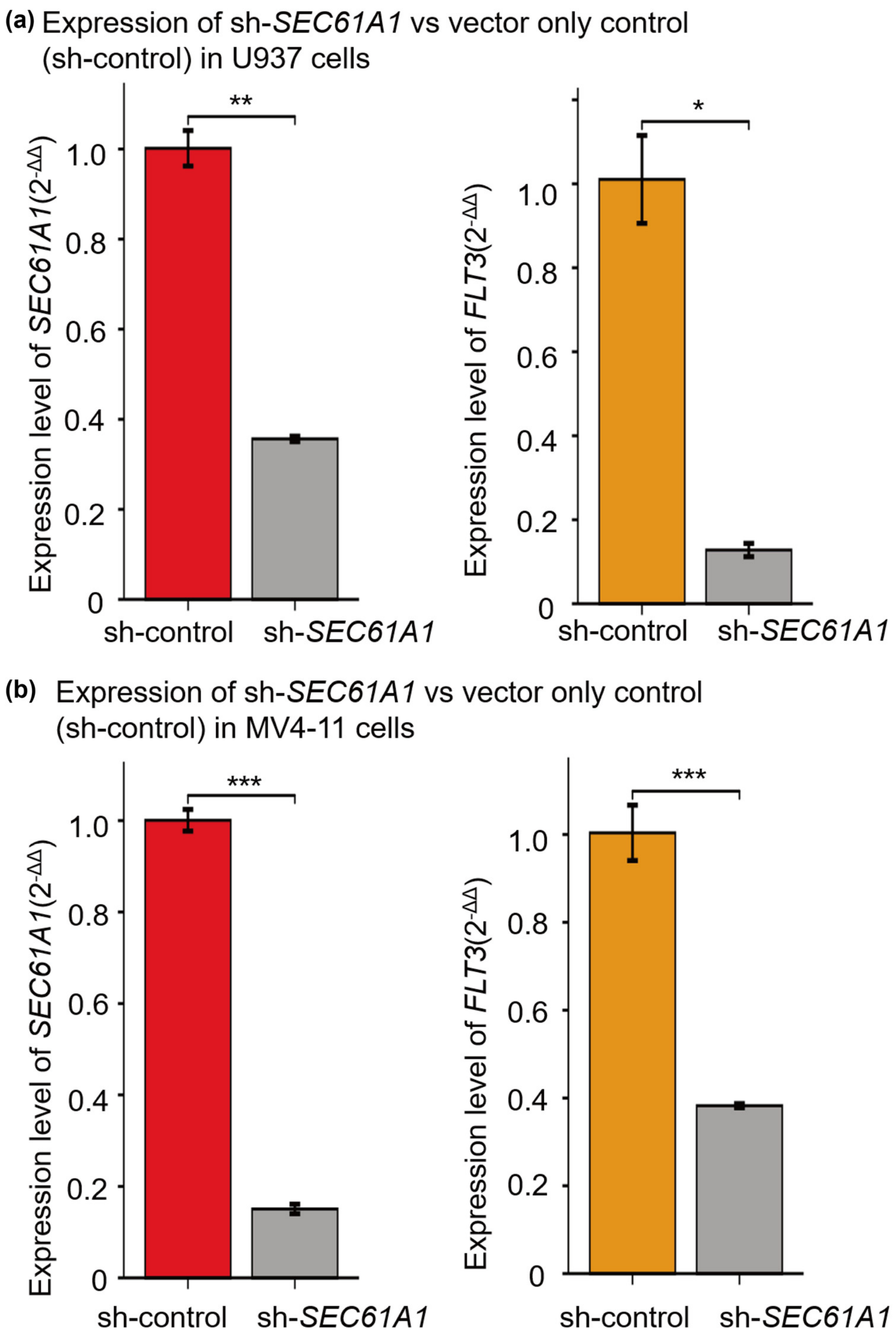

Figure S1: Knockdown efficiency of sh-*SEC61A1* in AML cells. Expression level of *SEC61A1* and *FLT3* in sh-*SEC61A1* *versus* sh-control group in U937 (A) and MV4-11 cell lines (B), respectively. *$P < 0.05$, **$P < 0.01$, ***$P < 0.001$.

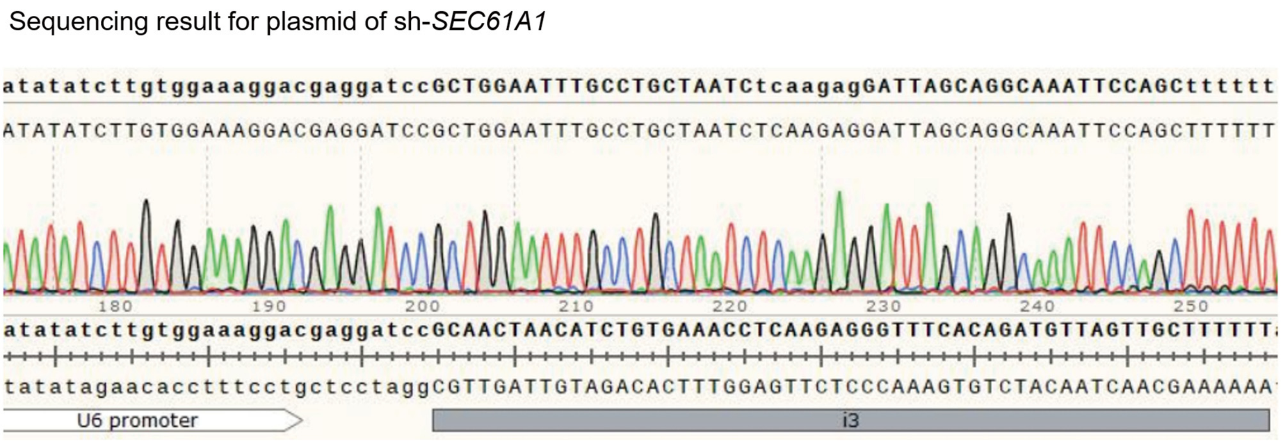

Figure S2: Sequencing for plasmid of sh-*SEC61A1* was validated by first generation sequencing.

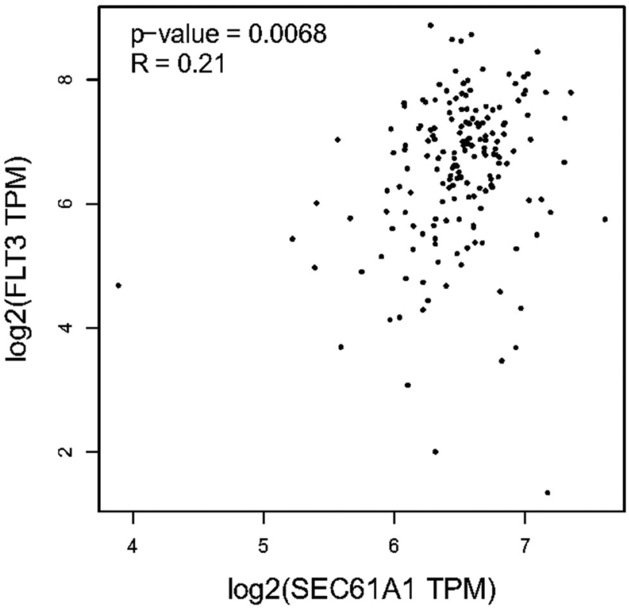

Figure S3: The correlation analysis of *SEC61A1* and *FLT3* expression in AML (TCGA).

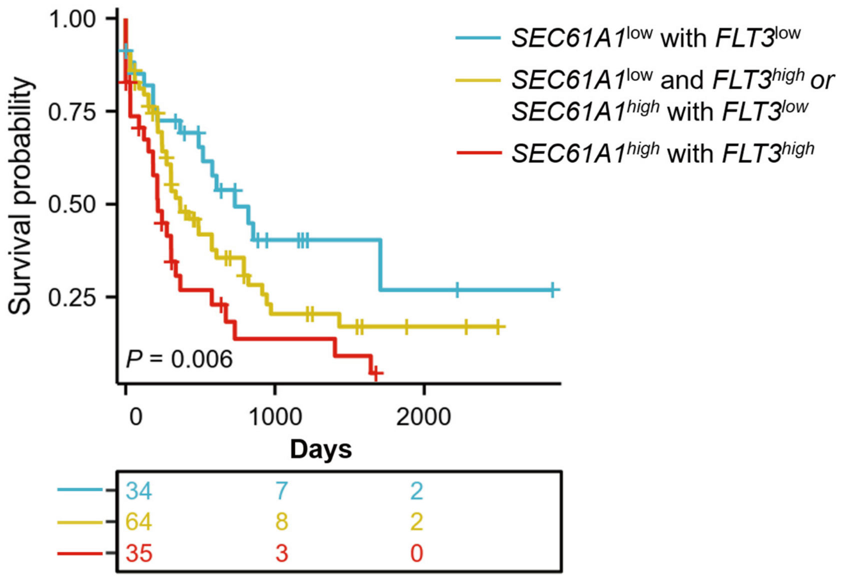

Figure S4: Survival analysis of non-M3 AML patients according to the *SEC61A1* and *FLT3* expression.
